# Comparative analysis of pathophysiological parameters between emphysematous smokers and emphysematous patients with COPD

**DOI:** 10.1038/s41598-019-57354-2

**Published:** 2020-01-15

**Authors:** Shuang Bai, Rui Ye, Cuihong Wang, Pengbo Sun, Li Zhao

**Affiliations:** Department of Pulmonary and Critical Care Medicine, Shengjing Hospital of China Medical University, Shenyang, Liaoning China

**Keywords:** Chronic obstructive pulmonary disease, Medical research

## Abstract

Emphysematous smokers with normal spirometry form a considerable proportion of the clinical population. However, despite presenting with respiratory symptoms and activity limitation, they cannot be diagnosed with chronic obstructive lung disease (COPD) according to current criteria. Thus, we aimed to determine whether emphysema in smokers has a different pathogenesis from that in patients with COPD. We compared 12 pairs of lung tissue samples from emphysematous patients with normal spirometry and COPD, and determined the degree of emphysema using computed tomography. With a focus on COPD-related pathogenesis, we independently assessed inflammatory response, protease-antiprotease balance, oxidative stress, and apoptosis in both groups. Both groups showed similar pathological changes at a comparable degree of emphysema; the expression of inflammatory factors was comparable, with overexpression of proteases and decreased levels of antiproteases. Moreover, there was no significant difference in the activities of glutathione and superoxide dismutase, and expression of apoptosis-related factors. In conclusion, emphysema in smokers with normal spirometry and in patients with COPD had similar pathogenesis. Forced expiratory volume in 1 second cannot be used as the sole diagnostic criterion in patients with COPD; early intervention is of great importance to such patients.

## Introduction

Chronic obstructive lung disease (COPD) is a chronic respiratory airway disease, with symptoms such as cough, sputum, and shortness of breath^1^. The two most prominent pathological changes associated with COPD are the structural destruction of lung tissue and airway remodelling^2^. Currently, spirometry is the sole diagnostic criterion for COPD^1,3^.

Forced expiratory volume in 1 second (FEV1) primarily reflects airflow obstruction and is therefore not suitable for assessing COPD symptoms such as emphysema^4–8^. Studies have shown that there is a poor correlation between FEV1 and COPD symptoms^5,6^, as well between FEV1 and the degree of emphysema, as assessed by computed tomography (CT)^7,8^.

Patients with COPD show high heterogeneity in terms of clinical symptoms, structural destruction, and airway damage^9,10^. In some patients, structural destruction of lung tissue is more prominent, and their emphysema severity develops progressively^11^. These patients are termed emphysema phenotype of COPD^12,13^, and their structure destruction can be assessed with chest CT scans^14,15^. However, chest CT scans have revealed that a considerable number of smokers with obvious emphysema and lung tissue damage show preserved pulmonary function^14–16^. According to the current diagnostic criteria, such emphysematous smokers cannot be diagnosed with COPD. If they have same pathogenesis as patients with emphysematous COPD, appropriate interventions to reduce the clinical symptoms and block emphysema progression would be challenging in emphysematous smokers^5,17^.

In this study, we hypothesized that emphysematous smokers with normal spirometry and emphysematous patients with COPD exhibit similar pathophysiological parameters. The appropriate diagnosis of emphysematous patients with normal spirometry cannot be achieved using only the ratio of forced expiratory volume in 1 second to forced vital capacity (FEV1/FVC)^5,17^, as it can delay their treatment and result in progression of structural destruction^11^. That will significantly affect the quality of life and survival time of patients^6–8,18^. To test this hypothesis, we collected lung tissue from emphysematous smokers with normal spirometry and emphysematous patients with COPD. Two groups of smokers and patients with COPD with similar emphysema scores were identified after screening. Finally, we examined the extent of inflammatory response, oxidative stress, protease-antiprotease balance, and apoptosis.

## Results

### Demographics of clinical subjects

**Figure 1:**
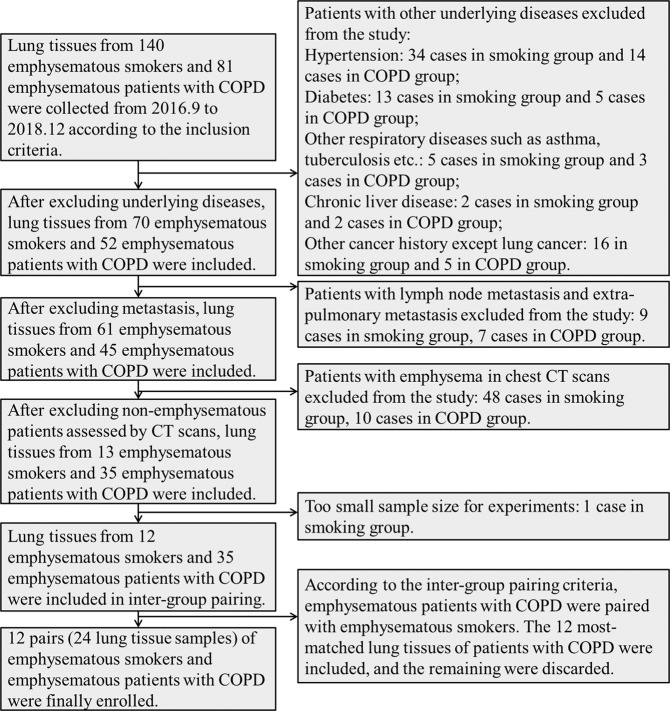
Process flowchart of screening and inter-group pairing.

We collected lung tissue from 140 emphysematous smokers and 81 emphysematous patients with COPD from 2016 to 2018, according to the inclusion criteria. After excluding subjects according to the exclusion criteria and CT emphysema score, only 13 emphysematous smokers with normal spirometry and 35 emphysematous patients with COPD were included in this study and categorized into the emphysematous smokers and emphysematous patient with COPD groups, respectively. According to the inter-group pairing criteria, 12 pairs (24 samples) of lung tissues were included in the experiment. The details of screening and inter-group pairing are shown in Fig. 1 and Table 1.

**Table 1:** Patient information related to inter-group pairing.

	Emphysematous smokers							Emphysematous patients with COPD					
ID	Sex	Age	Pathological type of tumour	Differentiation degree of tumour	Position of sampling	Years of smoking	ID	Sex	Age	Pathological type of tumour	Differentiation degree of tumour	Position of sampling	Years of smoking
S1	M	61	Squamous carcinoma	Low	Upper lobe of left lung	40	C1	M	66	Squamous carcinoma	Low	Upper lobe of right lung	50
S2	M	52	Adenocarcinoma	High	Upper lobe of right lung	40	C2	M	51	Adenocarcinoma	High	Upper lobe of left lung	30
S3	M	65	Adenocarcinoma	Low	Upper lobe of right lung	40	C3	M	58	Adenocarcinoma	Low	Upper lobe of right lung	40
S4	M	55	Adenocarcinoma	High	Upper lobe of left lung	30	C4	M	70	Adenocarcinoma	High	Lower lobe of left lung	40
S5	M	67	Adenocarcinoma	Low	Lower lobe of left lung	30	C5	M	60	Adenocarcinoma	High	Lower lobe of left lung	40
S6	M	64	Adenocarcinoma	Low	Lower lobe of right lung	40	C6	M	54	Adenocarcinoma	Low	Lower lobe of right lung	50
S7	M	63	Adenocarcinoma	Middle	Lower lobe of left lung	47	C7	M	56	Adenocarcinoma	High	Upper lobe of left lung	20
S8	M	54	Squamous carcinoma	Middle	Upper lobe of left lung	30	C8	M	51	Squamous carcinoma	Low	Upper lobe of right lung	30
S9	M	75	Squamous carcinoma	Middle	Lower lobe of right lung	10	C9	M	66	Squamous carcinoma	Middle	Lower lobe of left lung	40
S10	M	71	Squamous carcinoma	Middle	Upper lobe of right lung	50	C10	M	69	Squamous carcinoma	Middle	Upper lobe of left lung	30
S11	F	72	Adenocarcinoma	High	Upper lobe of right lung	20	C11	M	54	Adenocarcinoma	Middle	Upper lobe of right lung	40
S12	F	54	Adenocarcinoma	High	Lower lobe of left lung	20	C12	F	74	Adenocarcinoma	High	Upper lobe of right lung	60

There were no significant differences in the demographics between both groups. For spirometry parameters, statistical difference was observed only for FEV1, FEV1%, FEV1/FVC between both groups (Table 2).

**Table 2:** Demographic and spirometric features.

	Emphysematous smokers	Emphysematous patients with COPD	P value
Patients, n	12	12	
Age, years	62.75 ± 2.236	60.75 ± 2.297	0.539
Gender			1.000
Male, n (%)	10 (83.3)	11 (91.7)	
Female, n (%)	2 (16.7)	1 (8.3)	
Smokers			1.000
Current, n (%)	11 (91.7)	10 (83.3)	
Ex-smoker, n (%)	1 (8.3)	2 (16.7)	
Years of smoking	33.08 ± 3.454	39.17 ± 3.128	0.205
Pack-years	37.71 ± 7.178	42.29 ± 5.818	0.625
Pulmonary function test before bronchodilator			
FVC, %	93.73 ± 2.8	86.78 ± 3.78	0.154
FVC	3.404 ± 0.24	3.341 ± 0.22	0.847
FEV1, %	90.83 ± 3.03	67.87 ± 4.96	0.0007
FEV1	2.597 ± 0.17	2.068 ± 0.19	0.047
FEV1/FVC	76.94 ± 1.48	61.25 ± 2.65	<0.0001
Pulmonary function test after bronchodilator			
FVC, %	—	89.93 ± 3.84	
FVC	—	3.46 ± 0.27	
FEV1, %	—	68.11 ± 5.55	
FEV1	—	2.076 ± 0.21	
FEV1/FVC	—	59.53 ± 3.25	
Pathological type of tumour			1.000
Adenocarcinoma, n (%)	8 (66.7)	8 (66.7)	
Squamous carcinoma, n (%)	4 (33.3)	4 (33.3)	
Differentiation degree			1.000
High differentiation, n (%)	4 (33.3)	5 (41.7)	
Middle differentiation, n (%)	4 (33.3)	3 (25.0)	
Low differentiation, n (%)	4 (33.3)	4 (33.3)	
Lymphatic metastasis, n (%)	0 (0)	0 (0)	1.000

Data represent the mean ± SEM of computed tomography (CT) parameters for all patients in each group.

COPD, chronic obstructive pulmonary disease; FEV1, forced expiratory volume in 1 second; FVC, forced vital capacity.

### Identification of comparable degree of emphysema between emphysematous smokers and emphysematous patients with COPD

**Figure 2:**
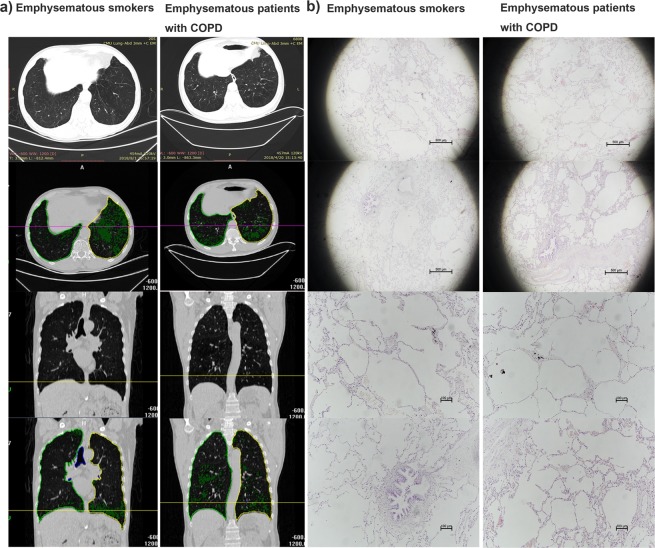
Representative chest CT images and HE images of lung sections. (**a**) Representative CT analysis images of emphysema in subjects. The left and right lungs are distinguished by surrounding lines of different colours (yellow, for left lung and green for right lung). Emphysema is represented by the green area in lungs. (**b**) Representative haematoxylin and eosin images of lung sections. Lung sections were stained with HE and examined under a light microscope. The magnification scale is indicated in each figure panel.

After preliminary screening by two experienced imaging specialists, the degree of emphysema in the subjects was analysed from chest CT scans using CT analysis software. The degree and distribution of emphysema were similar in both groups (Fig. 2). Among all analysed parameters, only the volume of air in emphysematous patients with COPD was slightly higher than that in emphysematous smokers (1951 ± 180.1 vs. 2525 ± 204.1, P = 0.047). Emphysema index (6.468 ± 2.14 vs. 8.936 ± 3.095, P = 0.513) and emphysema percentile density (−907.8 ± 10.95 vs. −930.4 ± 10.17, P = 0.149) were not statistically different between both groups (Table 3).

**Table 3:** Comparison of radiological features between both groups.

	Emphysematous smokers (n = 12)	Emphysematous patients with COPD (n = 12)	P value
% of air	81.08 ± 1.405	84.23 ± 0.8541	0.077
Volume of air (cm^3^)	1951 ± 180.1	2525 ± 204.1	0.047
% of tissue	18.92 ± 1.405	15.77 ± 0.8541	0.076
Volume of tissue (cm^3^)	423.5 ± 21.37	456.2 ± 22.11	0.300
Emphysema index, %	6.468 ± 2.14	8.936 ± 3.095	0.513
Emphysema percentile density (HU)	−907.8 ± 10.95	−930.4 ± 10.17	0.149

Data represent the mean ± SEM of computed tomography (CT) parameters for all patients in each group.

COPD, chronic obstructive pulmonary disease.

Additionally, haematoxyline-eosin (HE) staining revealed typical histological morphology of emphysema, such as obvious alveolar septal destruction, alveolar fusion, and pulmonary bullae formation, in both groups. The degree of pathological emphysema was also similar (Fig. 2).

### Expression of inflammatory factors in emphysematous smokers and emphysematous patients with COPD patients is similar

**Figure 3:**
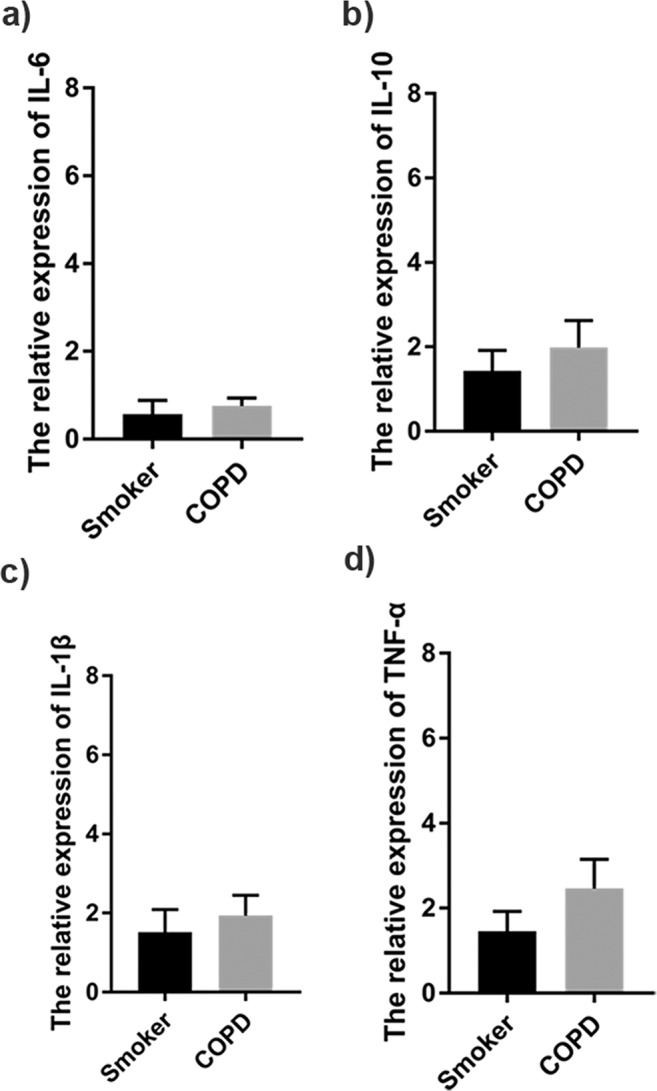
Expression of inflammatory factors in lung tissues. (**a**) IL-6 (n = 4 in each group), (**b**) IL-10 (n = 7 in each group), (**c**) IL-1b (n = 8 for smokers and 7 for patients with COPD), and (**d**) TNF-α (n = 7 for each group) mRNA expression in lung tissues was measured using real-time PCR. Data represent the mean ± SEM from three independent experiments.

There was no statistical difference in the expression levels of interleukin (IL)−6 (Fig. 3a; 0.57 ± 0.32 vs. 0.76 ± 0.18, P = 0.686), IL-10 (Fig. 3b; 1.43 ± 0.49 vs. 1.98 ± 0.64, P = 0.507), IL-1β (Fig. 3c; 1.52 ± 0.58 vs. 1.94 ± 0.51, P = 0.336), and tumour necrosis factor (TNF)-α (Fig. 3d; 1.46 ± 0.47 vs. 2.46 ± 0.69, P = 0.234), in lung tissue between both groups. However, the expression of inflammatory factors in the lung tissue of patients with COPD was higher than that in emphysematous smokers.

### Protease-antiprotease imbalance exists in both emphysematous smokers with normal spirometry and emphysematous patients with COPD

**Figure 4:**
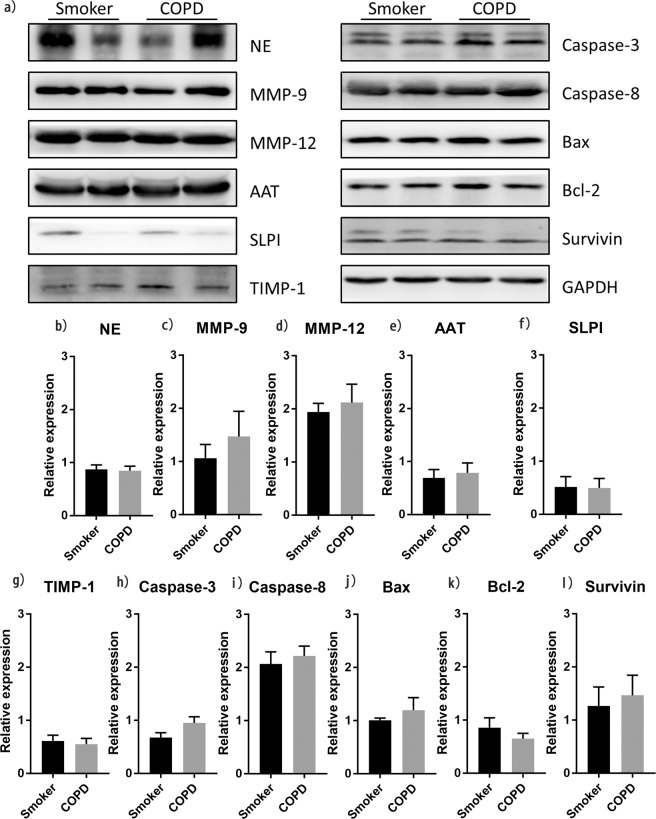
Comparison of protease-antiprotease balance and expression of apoptotic factors between both groups. Western blot analysis of (**a,b**) NE (n = 11 in each group), (**a,c**) MMP-9 (n = 11 in each group), (**a,d**) MMP-12 (n = 11 in each group), (**a,e**)AAT (n = 11 in each group), (a,f) SLPI (n = 11 in each group), (**a,g**) TIMP-1 (n = 11 in each group), (**a,h**) Caspase-3 (n = 11 in each group), (a,i) Caspase-8 (n = 11 in each group), (**a,j**) Bax (n = 11 in each group), (**a,k**) Bcl-2 (n = 11 in each group) and (**a,l**) Survivin (n = 11 in each group) expression in lung tissues of emphysematous smokers and emphysematous patients with COPD. There was no significant difference between both. Data represent the mean ± SEM from three independent experiments. Full-length blots are presented in Supplementary Fig. S1.

We determined the expression of representative factors that have opposing effects on the protease-antiprotease balance. There was no significant difference in the expression of neutrophil elastase (NE) (Fig. 4a,b; 0.87 ± 0.09 vs. 0.85 ± 0.08, P = 0.864), matrix metalloproteinase- 9 (MMP-9) (Fig. 4a,c; 1.07 ± 0.26 vs. 1.47 ± 0.47, P = 0.652) and MMP-12 (Fig. 4a,d; 1.94 ± 0.16 vs. 2.12 ± 0.35, P = 0.949) in lung tissue between both groups. Moreover, the expression of alpha-l antitrypsin (AAT) (Fig. 4a,e; 0.69 ± 0.16 vs. 0.78 ± 0.19, P = 0.693), secretory leukocyte peptidase inhibitor (SLPI) (Fig. 4a,f; 0.52 ± 0.19 vs. 0.50 ± 0.17, P = 0.945) and metalloproteinase −1 (TIMP-1) (Fig. 4a,g; 0.61 ± 0.11 vs. 0.55 ± 0.11, P = 0.721)—important anti-protease factors—were comparable in both groups.

### Expression of apoptosis-related factors in emphysematous smokers and emphysematous patients with COPD is similar

The expression of apoptosis-related factors Caspase-3 (Fig. 4a,h; 0.68 ± 0.09 vs. 0.95 ± 0.12, P = 0.079), Caspase-8 (Fig. 4a,i; 2.07 ± 0.23 vs. 2.22 ± 0.19, P = 0.519), and Bax (Fig. 4a,j; 1.01 ± 0.04 vs. 1.20 ± 0.24, P = 0.748) was similar between both groups. Moreover, the expression of anti-apoptotic molecules Bcl-2 (Fig. 4a,k; 0.86 ± 0.19 vs. 0.65 ± 0.10, P = 0.519) and Survivin (Fig. 4a,l; 1.27 ± 0.11 vs. 1.47 ± 0.11, P = 0.217) was similar between both groups.

### Emphysematous smokers and emphysematous patients with COPD exhibit similar levels of oxidative stress

**Figure 5:**
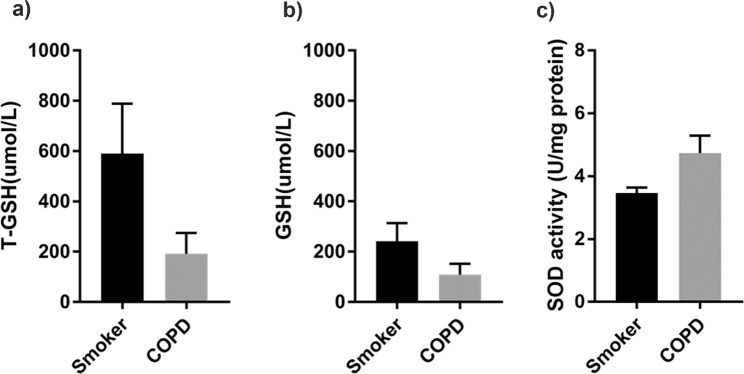
Measurement of GSH level and SOD activity. (**a**) T-GSH expression (n = 4 in each group), (**b**) GSH expression (n = 4 in each group), and (**c**) SOD activity (n = 4 in each group) in lung tissues of both groups are similar. Data represent the mean ± SEM from three independent experiments.

We used an oxidative stress kit to detect oxidative stress response in both groups. The major indicators of oxidative stress included total glutathione (GSH), reduced GSH, and superoxide dismutase (SOD) activity. The levels of total GSH (Fig. 5a; 590.6 ± 197.8 vs. 190.9 ± 83.49, P = 0.20) and reduced GSH (Fig. 5b; 241.6 ± 72.43 vs. 108.4 ± 43.36, P = 0.20) were not significantly different between both groups, and SOD activity (Fig. 5c; 3.46 ± 0.18 vs. 4.74 ± 0.56, P = 0.142) was comparable. However, the oxidative stress-related index for the COPD group was higher than that for the normal spirometry group.

## Discussion

COPD is a remarkably heterogeneous disease. CT identifying emphysema phenotype is one of the most important COPD sub-phenotypes. However, CT scan analysis has revealed that approximately 20% of the emphysematous smokers show preserved pulmonary function^19^. They might have significant respiratory symptoms (COPD Assessment Test scores ≥10), limitation of activity, and exacerbations of COPD; however, they did not meet the spirometric criteria of COPD^20^. More importantly, several studies have confirmed that emphysema is an independent risk factor for patient mortality, and is independent of airflow limitation^7^. Smokers without spirometrically diagnosed COPD have been overlooked in clinical practice^5,21,22^. Therefore, emphysematous smokers with normal spirometry should receive early intervention to prevent progression of structural destruction, improve their quality of life, and prolong their survival time^5–8,23–25^. Based on these clinical findings, we selected smokers with normal spirometry and patients with COPD showing a similar degree of emphysema, and determined inflammatory response, protease-antiprotease balance, oxidative stress levels, and apoptosis-related protein expression in their lung tissues, to identify pathophysiological differences between them.

Our results showed that the expression of IL-6, IL-10, IL-1β, and TNF-α did not significantly differ between both groups, indicative of comparable inflammatory response. The expression of IL-6 was lower than that of other inflammatory factors, which confirmed that the levels of inflammatory factors in lung tissue are different^26^. However, the expression of inflammatory factors in emphysematous patients with COPD was slightly higher than that in emphysematous smokers. This result is consistent with the view that smokers with high inflammatory levels are more likely to develop COPD^26^. NE, MMP-9 and MMP-12 are important protease family members, and AAT, SLPI and TIMP-1 plays an important role as an anti-protease^9,27,28^. The expression of these molecules was similar in both groups, suggesting that there is a significant protease-antiprotease imbalance in emphysematous smokers with normal spirometry. In addition, the level of reduced GSH was higher and SOD activity was lower in emphysematous smokers with normal spirometry than that in emphysematous patients with COPD. However, the difference was not statistically significant. We presume that oxidative stress might be one of the main reasons that caused differences in spirometry between both groups.

In order to perform comprehensive comparison, we assessed the expression of apoptosis-related proteins in both groups. The expression of Caspase-3, Caspasse-8, Bax, Bcl-2, and Survivin was not significantly different between both groups. However, the expression of Survivin in patients with COPD was relatively higher than that in emphysematous smokers, which is in contrast to the anti-apoptotic effect of Survivin. We speculate that the increased expression of Caspase family members might upregulate Survivin expression^29^. Moreover, the expression of Bcl-2, another anti-apoptotic factor, was relatively higher in emphysematous smokers than that in emphysematous patients with COPD. Studies have confirmed Bcl-2 expression does not correlate with Survivin expression, which may also explain the relatively higher expression of Survivin in patients with COPD^30^.

Our study demonstrates that smokers with normal spirometry and patients with COPD exhibit comparable inflammatory response, oxidative stress, protease-antiprotease balance, and apoptosis levels, under similar degree of emphysema. These results indicate that both groups of subjects exhibit similar pathophysiological parameters and therefore should be treated equally.

Previous studies have indicated that there is considerable proportion of emphysematous smokers with normal spirometry in clinical practice^12,14,16^. Emphysematous smokers develop more serious respiratory symptoms, have worse quality of life, and shorter survival time than other smokers^5–7^. However, a majority of these studies were clinical researches. In this study, we explored the intrinsic relationship between emphysematous smokers and emphysematous patients with COPD by examining major pathophysiological parameters, such as key molecules in inflammatory response, protease-antiprotease imbalance, oxidative stress, and apoptosis^31^. Our findings confirmed that both groups exhibit similar pathophysiological parameters. It indicated that emphysema patients with normal spirometry not only have structural destruction of lung tissue, but also had the pathological basis and disease development direction consistent with emphysema COPD patients.

As the emphysema sites might affect patient’s airflow diversely, both groups of patients revealed consistent pathological manifestations and variant airflow limitation degrees^32^. In addition, emphysematous patients in whom small airways are affected would be less sensitive to spirometry^33^, because FEV1 predominantly reflects the function of large airways. Consequently, FEV1 cannot determine the symptoms and severity of patients with COPD^4–6^. Hence, FEV1 result is not a sensitive indicator of emphysema and cannot be relied on to determine whether patients should receive intervention.

Using the pathogenesis of COPD and emphysema as the breakthrough point, we confirmed the close relation between emphysematous smokers and emphysematous patients with COPD at the molecular mechanism level, and provided strong evidence regarding similarities in the pathophysiological parameters and development direction between both groups, which compensate for inadequate in clinical trials. In addition, we analysed the degree of emphysema in terms of pathology and iconography. Image analysis software was used to analyse the degree of emphysema and to improve the accuracy of inter-group pairing. Considering the influence of systemic system on body fluids such as blood and sputum, we selected lung tissue as the experimental specimen to reflect the pathogenesis of emphysema in lungs. For the question of whether emphysematous patients with normal spirometry are really “disease-free” and “no treatment required”, we answered this question from a new perspective other than clinical research.

As COPD is not an indication for lobectomy, we strictly followed the criterion that only normal tissue located at more than 5 cm from the lesion site can be collected^34,35^. We performed strict inter-group matching in both groups to minimize the impact of the underlying disease on the experimental results. More than two respiratory physicians participated in history taking of the subjects to ensure the accuracy and credibility of the data.

This study has several limitations. Firstly, only 24 patients were included in the study because of the strict inclusion criteria and inter-group matching. Secondly, oxidative stress results may have been affected as the specimens were collected over 2 years for this study. In the future, the sample size may be increased to improve the accuracy of results. In addition, a recently published study confirmed that smoking-induced humoral immune mechanisms are involved in lung parenchymal damage, and B-cell may play an important role in the formation of emphysema. Therefore, relevant indicators should be considered in future experiments^19^.

In conclusion, emphysematous smokers that did not have significant airflow limitation showed similar pathophysiological characteristics to emphysematous patients with COPD, confirming the consistency of the pathogenesis and development direction of disease in both groups. Therefore, clinicians should relax the FEV1 criteria for emphysematous smokers with normal spirometry, and not consider them disease-free. In the future, attention must be given to early intervention for emphysematous smokers with normal spirometry to prevent progression of structural destruction, which ultimately affects their quality of life and survival time.

## Methods

### Subjects

Subjects were screened strictly according to the inclusion criteria and were grouped according to inter-group pairing.

The inclusion criteria were as follows:

(1) age of 40 to 80 years old; (2) a history of 10 or more pack-years of smoking; (3) patients with lung cancer preparing for chest surgery; (4) signed informed consent.

The exclusion criteria were as follows:

(1) bronchial asthma, bronchiectasis, active tuberculosis, or other respiratory diseases; (2) glomerulonephritis, nephrotic syndrome, Ig-A nephropathy, renal failure, or other chronic kidney diseases; (3) autoimmune liver disease, acute or chronic hepatitis, cirrhosis, ulcerative colitis, Crohn’s disease, and other chronic digestive diseases; (4) endocrine system diseases such as diabetes, hyperthyroidism, thyroiditis, or Graves’ disease; (5) various connective tissue diseases; (6) essential hypertension or primary pulmonary hypertension; (7) presence of tumour in the past or present besides lung cancer; (8) tumour had metastasized in lymph nodes or outside the lungs; (9) treated with steroids or other immunosuppressive agents for various reasons; and (10) haemophilia, hereditary spherocytosis, or other genetic diseases.

In order to ensure that the degree of emphysema is consistent between both groups, and reduce tissue heterogeneity caused by underlying diseases and surgical diseases, the following inter-group pairing criteria was implemented to screen specimens:

(1) showed a comparable degree of emphysema in chest CT scans; (2) same gender; (3) age difference of less than 10 years; (4) identical pathological tumour type; (5) similar differentiation degree of tumour; (6) similar position of sampling (accurate to the lobes of lungs); and (7) similar number of smoking pack-years.

Emphysema in chest CT scans was judged by two experienced imaging specialists and the degree of emphysema was validated using CT analysis software^23,24^. Comprehensive inquiries and spirometry tests using standard spirometric techniques were conducted by two respiratory physicians^36^.

Patients with COPD were diagnosed for respiratory symptoms, such as cough, sputum, and breathlessness, and those showing an FEV1/FVC ratio of <0.7 after short acting bronchodilator inhaled^1^. Emphysematous smokers were defined as current smokers with normal spirometry and emphysema detected in CT imaging.

This study was performed in accordance with the Declaration of Helsinki. The protocol for this study was approved by the Ethics Committee of Shengjing Hospital of China Medical University (Shenyang, China; ethical no.2016PS342K). Written informed consent was obtained from each participant prior to obtaining tissue samples and chest CT scans.

### Image analysis for assessing the degree of emphysema

Chest CT scans at full inspiration were performed for all subjects using Philips Ingenuity Core 128 CT scanner (Philips, the Best, the Netherlands) or TOSHIBA Aquilion ONE (Toshiba, Tokyo, Japan). All images were acquired with the following parameters: tube voltage, 120 kV; tube current, 180 mA; slice thickness, 3 mm; and reconstruction matrix, 512 × 512. The details regarding CT acquisition parameters are listed in Supplementary Table S1. All patients were scanned craniocaudally in the supine position. Two radiologists performed the radiological measurements. Chest CT raw data sets were obtained from the CT workstation (Neusoft, Shenyang, China) and analyzed using Pulmonary Toolkit (PTK) in Matlab (R2016a) (The MathWorks, Inc., Natick, MA, USA)^37^. The analysis results were validated by visualization with NeuLungCARE in BW CT workstation (Neusoft, Shenyang, China). We employed a rule that when the CT value of a pixel is less than −950 HU, the software will determine the voxel as emphysema area. The emphysema index was determined as volume fraction of the lungs below −950 HU at full inspiration^38,39^. Emphysema percentile density was determined as the 15^th^ percentile lung density (PD15) derived from the CT voxel distribution histogram of whole lung^40^. All CT images were input into the software in the form of raw data, and analysis results were obtained. Data analysis was completed using PTK. Visualization of the degree of emphysema and emphysema distribution were performed using NeuLungCARE.

### Pulmonary function test

Spirometry parameters were measured in the subjects using the Medgraphics Platinum Elite DL Plethysmograph (St. Paul, Minneapolis, MN, USA). Pulmonary function tests were conducted by two respiratory physicians, and the results of measurement met the requirement of the American Thoracic Society. In brief, a minimum of three and a maximum of eight tests of lung function were performed for each subject, and there were more than two acceptable tests with repeatability within 150 ml. Acceptable tests were defined as follows: No medications in the past 24 h, subject exhaled quickly without hesitation, and showed explosive power. A good start of exhalation with extrapolated volume was defined as <5% of FVC or 150 ml, whichever was greater. The expiratory time was ≥6 s, and the expiratory platform (volume change <25 ml/s) in the time volume curve appeared for more than 1 s. The best value of FEV1, FVC in spirometry tests of each patient was taken. At an FEV1/FVC ratio <0.7, the subjects were administered with 400 mcg albuterol, reversibility tests were conducted after 15 min.

### Preparation of lung tissue specimens

The lung tissues of subjects were prepared as previously described^34,35^. In brief, lung tissue specimens were excised as far as possible from the tumour (at least 5 cm from the lesion site). The specimens appeared as normal tissue. The location (peripheral tissue extending up to the pleural surface) and amount (~1 cm³) of each sample was standardized to ensure approximately equal amounts of lung parenchyma, small airways, and pulmonary vessels in specimens. After collection, lung tissue specimens were washed with normal saline and dried with sterile gauze to remove residual blood. Specimens were immediately frozen in liquid nitrogen and stored at −80 °C until use.

### HE staining

HE staining was conducted according to routine protocols^41^. Briefly, lung tissues were dissected, fixed in 4% paraformaldehyde, embedded with paraffin after dehydration, and cut into 2.5-µm thick cross-sections. The paraffin-embedded sections were subjected to HE staining to visualize under light microscopy (Nikon, Japan) for lung morphology and Nis-Elements F3.0 (Nikon, Japan) software was used as image acquisition tool. The remaining tissue specimens were quickly frozen in liquid nitrogen for the analysis of protein and RNA expression.

### Western blot analysis

Proteins were extracted from lung tissues and quantified using the BCA protein concentration assay kit (Solarbio, China). Equal amounts of protein were resolved on sodium dodecyl sulphate-polyacrylamide gel electrophoresis gels, and transferred to polyvinylidene fluoride membranes. The membranes were blocked with 5% non-fat milk in TBST (10 mM Tris-HCl pH 7.4, 100 mM NaCl, 0.5% Tween-20) for 2 h at room temperature and incubated overnight at 4 °C with primary antibodies. The following primary antibodies were used according to the manufacturer’s protocol: anti-MMP9 (ab76003), anti-TIMP1 (ab109125), and anti-Caspase3 (ab32351) from Abcam (Cambridge, Cambridgeshire, UK); anti-Bax (#2772) from Cell Signaling Technology (Danvers, MA, USA); anti-GAPDH from Proteintech (Chicago, IL,USA); anti-MMP12 (bs-1854R) from Bioss (Beijing, China), Anti-Caspase8(WL02434), anti-Bcl2 (WL01556), and anti-Survivin (WL01684) from Wanleibio (Shenyang, Liaoning, China). The membranes were washed in TBST and incubated with secondary antibodies for 1.5 h. After extensive washing, the membranes were visualized using enhanced chemiluminescence reagent. Final images were analyzed using ImageJ software.

### Quantitative reverse transcriptase polymerase chain reaction

Total RNA was extracted from lung tissues using the RNAiso reagent (TaKaRa, Naha, Japan), according to the manufacturer’s protocol. RNA was reverse transcribed to cDNA using the TaKaRa reverse transcription kit. Finally, cDNAs were amplified and detected using the SYBR® Green PCR Kit (Takara, Naha, Japan) with a Roche 480 Real-Time PCR System (Roche, Basel, Switzerland). The amplification parameters were as follows: pre-incubation for 5 min at 95 °C, followed by 40 cycles of initial denaturation at 95 °C for 5 s and annealing at 60 °C for 30 s. Glyceraldehyde phosphate dehydrogenase (GAPDH) was used as reference gene. No signal was detected in the negative control (no template). The following primer sequences were used: IL-6, forward 5′-ACCCCCAATAAATATAGGACTGGA-3′, reverse 5′-GAGAAGGCAACTGGACCGAA-3′; IL-10, forward 5′-CAGAAGTACCTGAGCTCGCC-3′, reverse 5′-AGATTCGTAGCTGGATGCCG-3′; IL-1β, forward 5′-GCCAGTGAAATGATGGCTTATT-3′, reverse 5′-AGGAGCACTTCATCTGTTTAGG-3′; TNF-α, forward 5′-GCTGCACTTTGGAGTGATCG-3′, reverse 5′-TCACTCGGGGTTCGAGAAGA-3′; and GAPDH, forward 5′-CCTGGTATGACAACGAATTTG-3′, reverse 5′-CAGTGAGGGTCTCTCTCTTCC-3′.

### Measurement of glutathione (GSH) and superoxide dismutase (SOD) levels

Total GSH and reduced GSH levels and SOD activity were measured using oxidative stress assay kits (Jiancheng, Nanjing, China), according to the manufacturer’s protocol.

### Statistical analysis

Statistical analyses were performed using SPSS 22.0 software (SPSS, Inc., Chicago, IL, USA) and GraphPad Prism 7 (GraphPad, La Jolla, CA, USA). Data of continuous variables were expressed as the mean ± standard error of mean. The Student’s *t*-test was used to compare means between groups of data with normal distribution, and the Mann-Whitney U test was used to compare means between groups of data with non-normal distribution. Fisher’s exact probability was used to compare data of categorical variables. P < 0.05 was considered statistically significant.

## Supplementary information

Supplementary Information 

Supplementary  Information 2

## Data Availability

The datasets used during the current study are available from the corresponding author on reasonable request.
