# Supplementary material for: Comparative analysis of pathophysiological parameters between emphysematous smokers and emphysematous patients with COPD
Source: Sci Rep. 2020 Jan 15;10:420. doi: 10.1038/s41598-019-57354-2 (PMC6962428; doi:10.1038/s41598-019-57354-2)
Supplement: Supplementary file 1 — Supplementary Information [file 41598_2019_57354_MOESM1_ESM.pdf]

Supplementary Figure S1

Comparative analysis of pathophysiological parameters between emphysematous smokers and emphysematous patients with COPD

Shuang Bai[1], Rui Ye[1], Cuihong Wang[1], Pengbo Sun[1], Li Zhao[1,*]

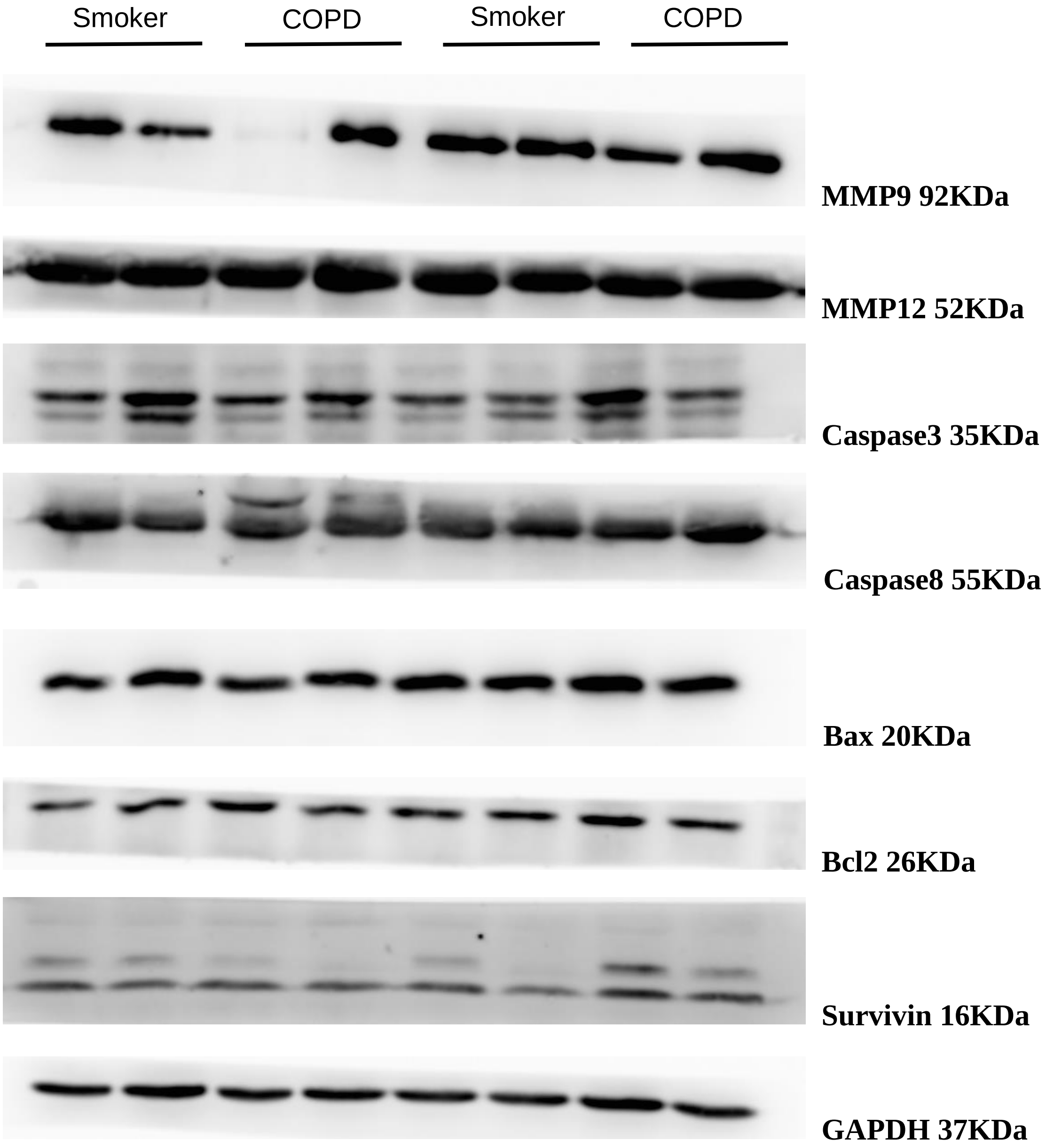

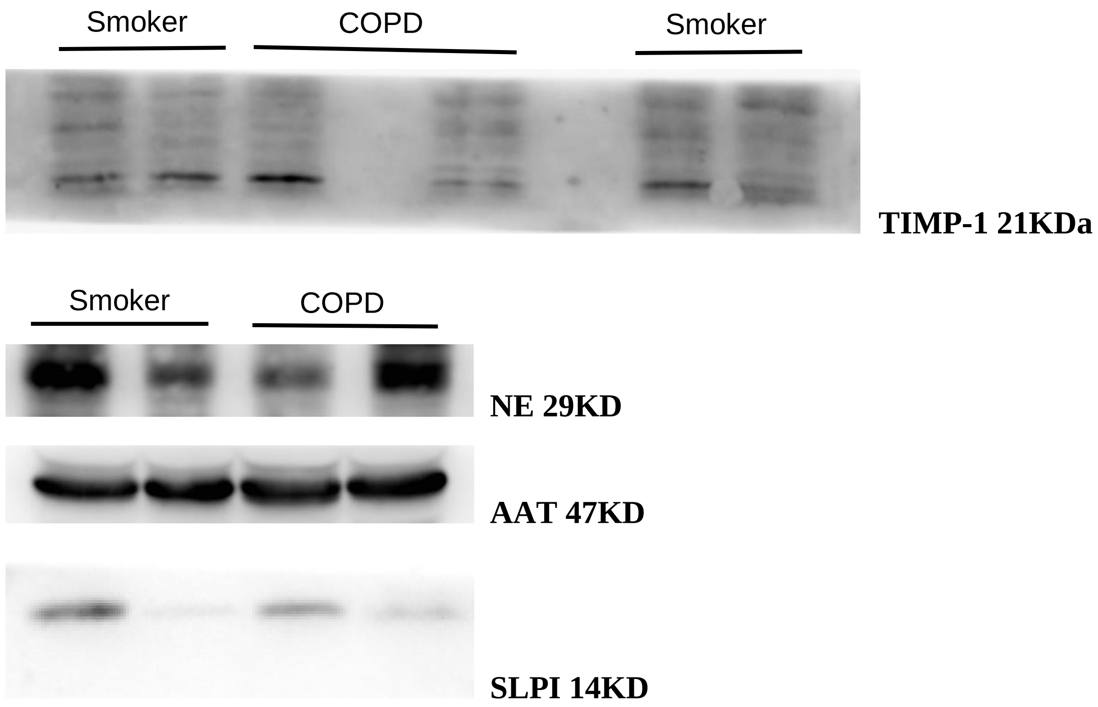
Smoker
COPD
Smoker
TIMP-1 21KDa
Smoker
COPD
NE 29KD
AAT 47KD
SLPI 14KD
