# Supplementary material for: Comparative analysis of pathophysiological parameters between emphysematous smokers and emphysematous patients with COPD
Source: Sci Rep. 2020 Jan 15;10:420. doi: 10.1038/s41598-019-57354-2 (PMC6962428; doi:10.1038/s41598-019-57354-2)
Supplement: Supplementary file 2 — Supplementary Information 2 [file 41598_2019_57354_MOESM2_ESM.pdf]

Supplementary Table S1

Comparative analysis of pathophysiological parameters between emphysematous smokers and emphysematous patients with COPD

Shuang Bai[1], Rui Ye[1], Cuihong Wang[1], Pengbo Sun[1], Li Zhao[1,*]

ID	FOV(mm)	Pixel size (mm)	Reconstruction diameter(cm)	Number of slices	CT scanner
S1	400.00	0.653	334	101	TOSHIBA Aquilion ONE
S2	500.00	0.844	432	169	Philips Ingenuity Core 128
S3	500.00	0.754	386	112	Philips Ingenuity Core 128
S4	500.00	0.688	352	155	Philips Ingenuity Core 128
S5	500.00	0.781	400	141	Philips Ingenuity Core 128
S6	500.00	0.738	378	110	Philips Ingenuity Core 128
S7	500.00	0.782	400	115	Philips Ingenuity Core 128
S8	500.00	0.764	391	112	Philips Ingenuity Core 128
S9	500.00	0.813	416	141	Philips Ingenuity Core 128
S10	500.00	0.699	358	125	Philips Ingenuity Core 128
S11	500.00	0.748	383	133	Philips Ingenuity Core 128
S12	500.00	0.949	486	135	Philips Ingenuity Core 128
E1	500.00	0.738	378	123	Philips Ingenuity Core 128
E2	500.00	0.773	396	152	Philips Ingenuity Core 128
E3	500.00	0.835	427	136	Philips Ingenuity Core 128
E4	500.00	0.893	457	151	Philips Ingenuity Core 128
E5	500.00	0.768	393	144	Philips Ingenuity Core 128
E6	500.00	0.684	350	135	Philips Ingenuity Core 128
E7	400.00	0.732	375	131	TOSHIBA Aquilion ONE
E8	500.00	0.769	394	112	Philips Ingenuity Core 128
E9	500.00	0.746	382	131	Philips Ingenuity Core 128
E10	500.00	0.760	389	134	Philips Ingenuity Core 128
E11	500.00	0.787	402	122	Philips Ingenuity Core 128
E12	500.00	0.713	365	120	Philips Ingenuity Core 128

Supplementary Table S1. Image scanning and reconstruction parameters of subjects.
